# Phosphodiesterase 3 inhibition and cough in elderly asthmatics

**DOI:** 10.1186/1745-9974-1-11

**Published:** 2005-11-24

**Authors:** Yoshihisa Ishiura, Masaki Fujimura, Kouichi Nobata, Miki Abo, Takayoshi Oribe, Shigeharu Myou, Hiroyuki Nakamura

**Affiliations:** 1 The Department of Internal Medicine, Toyama City Hospital, Toyama, Japan; 2 Respiratory Medicine, Cellular Transplantation Biology, Kanazawa University Graduate School of Medicine, Kanazawa, Japan

**Keywords:** cough reflex sensitivity, capsaicin, cilostazol, phosphodiesterase, bronchial asthma

## Abstract

**Aims:**

Cough is a common symptom of bronchial asthma, a chronic inflammatory airway disease. Recently, the therapeutic effects of selective phosphodiesterase (PDE) inhibitors have been focused on bronchial asthma. This study was designed to investigate the clinical effect of PDE 3 inhibition on cough reflex sensitivity in elderly patients with bronchial asthma.

**Methods:**

Effects of cilostazol, a PDE 3 inhibitor, on cough response to inhaled capsaicin were examined in 11 patients over 70 years with stable asthma in a randomized, placebo-controlled cross over study. Capsaicin cough threshold, defined as the lowest concentration of capsaicin eliciting five or more coughs, was measured as an index of airway cough reflex sensitivity.

**Results:**

The cough threshold was significantly (p < 0.05) increased after two-week treatment with cilostazol (100 mg twice a day orally) compared with placebo [48.8 (GSEM 1.4) vs. 29.2 (GSEM 1.3) μM].

**Conclusion:**

These findings indicate that PDE 3 inhibition may be a novel therapeutic option for elderly patients with asthma, especially for their cough symptoms.

## Introduction

Chronic cough is a frequent problem in general practice and one of the commonest reasons for referral to respiratory clinic. A patient's quality of life becomes severely affected through loss of sleep, interruption of work and social embarrassment. Every effort should be made to clarify the cause of cough because specific therapy has a higher likelihood of success than empirical therapy. A previous study revealed that patients with persistent cough had three times the risk of developing chronic wheezing as compared to normal subjects [2]. Thus, it is important to disclose the mechanism of persistent cough and to develop more efficacious treatment. Though cough has been considered to result from stimulation of airway sensory nerve endings within the respiratory tract [1], the potential mechanism by which the cough reflex may be altered in humans remains obscure.

Recently, considerable attention has been focused on the potential use of selective inhibitors of cyclic nucleotide phosphodiesterases (PDEs) in the treatment of respiratory diseases as PDE isoenzymes may play an important role in the regulation of airway caliber and bronchial smooth muscle function [3]. It has been shown that PDE 3 and PDE 4 are the major adenosine 3' 5'-cyclic monophosphate (cyclic-AMP) – hydrolyzing enzymes and that human airway smooth muscle contains isozymes of the PDE families [4,5]. Furthermore, human lung tissue contains multiple PDE isozymes [6]. Therefore, it is important to determine the possible role of inhibition of these PDE isozymes *in vivo*. Though previous research failed to prove a bronchodilator effect of a PDE 3 and PDE 4 dual inhibitor, zardaverine, in patients with partially reversible chronic airway obstruction [7], others indicated the protective effect of selective PDE 3 and PDE 4 inhibitors [8,9]. We have demonstrated that a phosphodiesterase 3 inhibitor, cilostazol, reduces bronchial hyperresponsiveness to inhaled methacholine in elderly patients with stable asthma [10].

Based on these findings, this study was designed to elucidate the potential importance of orally administered cilostazol on cough reflex sensitivity to inhaled capsaicin in asthmatic elderly patients.

## Subjects and Methods

### Subjects

Eleven patients over 70 years with stable bronchial asthma (4 males and 7 females) with a mean age of 74.9 ± 1.3 (± SEM) (range 70–81) yrs participated in this study. All patients were lifetime nonsmokers or ex-smokers with no history of viral infection for at least 4 weeks prior to the study. Informed consent was obtained from all subjects. Characteristics of individual patients are shown in table 1. This study was approved by the Ethics Committee of our hospital.

Each asthmatic patient satisfied the American Thoracic Society definition of asthma, with symptoms of episodic wheezing, cough, shortness of breath responding to bronchodilators and reversible airflow obstruction documented on at least one previous pulmonary function study [11]. Reversibility was defined as greater than 12% increase in the forced expiratory volume in one second (FEV_1_) following inhalation of 200 μg salbutamol sulfate. All patients had bronchial hyperresponsiveness as shown in table 1 and were taking oral (short-acting clenbuterol) and/or aerosol β_2_-agonists (short-acting procaterol), inhaled steroids (beclomethasone dipropionate) and/or mucolytic agents (carbocysteine). They had not received oral theophylline or oral steroid therapy for at least eight weeks. This study was carried out when their symptoms were mild and stable.

**Table 1:** Clinical characteristics of asthmatic patients

											Treatment			
											
Patient number	Age (yr)	Sex	Height (cm)	Type	Severity	Total IgE in serum (IU/ml)	Specific IgE in serum	Complication of allergic disease	RT20-FEV1 (mg/ml)*	Bronchodilator response (%)**	BDP (μg/day)	Theophylline (mg/day)	Clenbuterol (μg/day)	Carbocysteine (mg/day)
1	81	M	154	Int	Moderate	75	-	-	2.50	34.0	800	0	40	1500
2	72	F	151	Ext	Moderate	464	HD	AR	2.50	25.4	800	0	0	0
3	80	F	143	Ext	Moderate	3	Mite, HD	-	1.25	27.0	800	0	40	0
4	72	F	148	Int	Mild	28	-	-	0.08	19.4	0	0	0	1000
5	79	F	142	Int	Moderate	37	-	-	5.00	27.6	800	0	20	1500
6	72	F	150	Int	Moderate	57	-	-	0.31	22.3	400	0	40	0
7	72	F	147	Ext	Moderate	647	HD, Ceder	-	0.31	31.8	800	0	20	1500
8	70	F	140	Int	Moderate	17	-	-	1.25	17.6	800	0	40	1500
9	75	M	162	Ext	Moderate	148	HD, Ceder	AR	2.50	14.1	800	0	40	1500
10	71	M	165	Ext	Moderate	133	Mite, Ceder	AR	1.25	14.6	0	0	40	1500
11	80	M	165	Int	Moderate	2	-	-	2.50	12.6	400	0	0	0

Ext, extrinsic; Int, intrinsic; HD, house dust; AR, allergic rhinitis; BDP, beclomethasone diproprionate inhalation.

*PC20-FEV1 shows concentration of inhaled methacholine causing a 20% fall in FEV1.

**Bronchodilator response means percent increase in forced expiratory volume in 1s (FEV1) from the baseline value after inhalation of 300 μg of salbutamol sulfate.

All patients used inhaled β2-agonists (salbutamol or procaterol) on demand.

### Assessment of cough reflex sensitivity to inhaled capsaicin

Cough reflex sensitivity was assessed by a capsaicin provocation test [12]. Capsaicin (30.5 mg) was dissolved in Tween 80 (1 mL) and ethanol (1 mL) and then dissolved in physiological saline (8 mL) to make a stock solution of 1 × 10^-2 ^M, which was stored at -20°C. This solution was diluted with physiological saline to make solutions starting at a concentration of 0.49 μM and increased by doubling concentrations up to 1000 μM. Each subject inhaled a control solution of physiological saline followed by progressively increasing concentrations of the capsaicin solution. Solutions were inhaled by the subjects for 15 s every 60 s, by tidal mouth-breathing whilst wearing a noseclip from a Bennett Twin nebulizer (3012-60 cc, Puritan-Bennett Co., Carlsbad, California, USA). Increasing concentrations were inhaled until five or more coughs were elicited. The nebulizer output was 0.21 mL/min. The number of capsaicin-induced coughs were counted by a blindfolded medical technician in our pulmonary function laboratory. The cough threshold was defined as the lowest concentration of capsaicin that elicited five or more coughs.

### Study protocol

The subjects' concomitant medication was stopped at 9.00 p.m. on the previous day to allow a washout time of 12 h or more before the measurement of cough threshold to inhaled capsaicin at 10.00 a.m. on each test day to reduce the diurnal variability of cough response.

Each patient attended 4 times, once every 2 weeks, at the same time each day. A control measurement of capsaicin cough threshold was carried out before the first treatment. After a two week wash out period, treatment with cilostazol and a placebo was performed in a randomized, cross-over fashion, with a washout period of 2 weeks between treatments. Two cilostazol tablets (100 mg) or their placebo were taken orally two times a day for 14 days at 8.00 a.m. on the test day. FEV_1 _was measured on a dry wedge spirometer (Transfer Test, P.K. Morgan Ltd., UK) before the capsaicin challenge to assess the bronchoactive effect of the treatment regimens.

### Data analysis

Capsaicin cough threshold values were expressed as a geometric mean with a geometric standard error of the mean (GSEM). Forced vital capacity (FVC) and FEV_1 _were shown as arithmetic mean values ± SEM. The cough threshold, the FVC and the FEV_1 _values were compared between each pair of the four test periods (run-in, placebo treatment, wash out and cilostazol treatment) by the Wilcoxon signed-ranks test. Data was transformed to logarithmic values for cough threshold at this test. A p-value of 0.05 or less was taken as significant.

## Results

**Figure 1:**
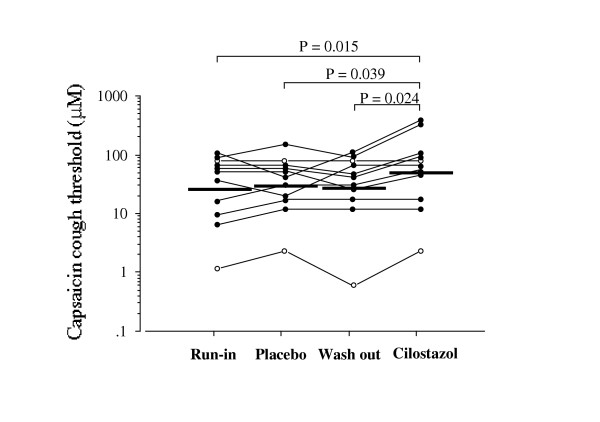
Individual data of capsaicin cough threshold before each treatment and after placebo and cilostazol treatments in elderly patients with stable bronchial asthma. Each horizontal bar represents geometric mean value. Closed circles and open circles represent patients undergoing steroid inhalation therapy and patients without steroid inhalation therapy, respectively. P values: Wilcoxon signed-ranks test using logarithmically transformed values.

Cough threshold to inhaled capsaicin before each treatment (run-in and washout period) and after treatment with cilostazol and placebo are shown in figure 1. Geometric mean values for the cough threshold were 25.9 (GSEM 1.4) μM in run-in period, 27.5 (GSEM 1.4) μM in washout period, 48.8 (GSEM 1.4) μM after cilostazol treatment and 29.2 (GSEM 1.3) μM after placebo treatment. The cough threshold after the cilostazol treatment was significantly (p < 0.05) greater than the value after the placebo treatment. FVC or FEV1 value was not significantly different between run-in period, washout period, cilostazol treatment and placebo treatment as shown in table 2.

There were no changes in serum IgE and peripheral blood eosinophils, therefore, treatment with cilostazol did not affect the IgE production or peripheral blood eosinophil count. After the administration of cilostazol, none of the patients complained of headache. Other adverse effects such as palpitations, flushing and dizziness were not observed with the cilostazol treatment in patients participating in this study, since the side effects of cilostazol are less frequent in elderly patients as shown in our previous study [10].

**Table 2:** Pulmonary function on cilostazol and placebo treatments in patients with bronchial asthma

	Run-in	Placebo	Wash out	Cilostazol
FVC as % pred. (%)	102.3 ± 5.7	104.7 ± 5.8	102.1 ± 5.1	108.4 ± 4.7
FEV1 as% pred. (%)	98.5 ± 8.2	100.4 ± 7.7	98.5 ± 6.6	106.3 ± 7.3

Data are shown as mean value ± standard error of the mean.

## Discussion

The present study showed that two-week treatment with a PDE 3 inhibitor, cilostazol, increased the cough threshold to inhaled capsaicin in elderly patients with stable asthma. No difference could be found in the baseline pulmonary function, peripheral blood eosinophil counts and IgE production between cilostazol and placebo treatments. From these findings, PDE 3 inhibition may be useful in elderly patients suffering from bronchial asthma, especially cough predominant asthma.

Although cough is a protective reflex that facilitates the expulsion of mucus from the airways, chronic cough causes major functional limitation in a great number of people who seek medical service. It is well known that cough can be the sole manifestation in some asthmatic patients [13], however, the precise mechanism correlating to the cough reflex in this disorder remains obscure. A recent study revealed that inflammatory mediators play major roles in the pathogenesis of bronchial asthma, however, the relationship between inflammatory mediators and airway cough reflex sensitivity also remains unclear. Previous studies showed that some inflammatory mediators can modulate the sensitivity of the cough reflex [14,15]. We indicated that intrinsic thromboxane A2 (TxA2) is a possible modulator, augmenting both airway cough reflex sensitivity and bronchial responsiveness whilst not having a bronchoconstricting effect in stable asthmatics [14,16,17]. Other studies reported that prostaglandin F2α (PGF2α) enhances airway cough reflex sensitivity with bronchoconstricting effect [18,19]. It has also been shown that inhaled prostaglandin E2 (PGE2), which acts as a bronchodilator, enhances cough reflex sensitivity [19,20]. These findings indicate the variable role of inflammatory mediators in the local control of cough reflex with no relation to bronchoconstriction.

Previous studies have shown the effects of selective inhibition of PDE isozymes in inhibition of inflammatory cell function and relaxation of airway smooth muscle in asthmatic airways [3-9,21]. Bachelet et al have shown that alveolar macrophages from asthmatic patients have increased PDE activity [22]. Other researchers have indicated that PDE 3 is closely coupled to the regulation of prostaglandin D2 (PGD2) generation [23]. Recently we demonstrated the bronchoprotective effect of PDE 3 inhibition in asthmatic patients [10,24,25], on the basis that PDE 3 is indeed present in human airway smooth muscle [26]. We, therefore, carried out this study on the assumption that PDE 3 activity in an asthmatic airway might also lead to increased sensitivity of airway cough response and concluded that a selective PDE 3 inhibitor, cilostazol, can modulate to reduce the airway cough sensitivity to inhaled capsaicin. We also showed that there was no improvement in lung function despite our previous study [10]. Though the precise mechanism for this discordant remains obscure, we stipulate that the difference in the cilostazol administration period may be a possible cause of the discordant, because in our previous study, bronchodilation was observed with a single administration of cilostazol. Precise mechanisms for the improvement of cough reflex sensitivity indicated in this study also remains unclear because we did not measure PC20. One of the possible mechanisms is that elevation of cyclic-AMP induced by PDE 3 inhibition may play some role in the regulation of cell activity and airway cough reflex sensitivity [26].

Furthermore, the bronchoprotective effect of PDE 3 inhibition for non-asthmatic subjects was not examined. There is therefore a need for further studies in patients with other bronchial disorders and normal subjects.

In conclusion, the present study clearly indicates that PDE 3 inhibition can attenuate cough reflex sensitivity in the airways of elderly asthmatic patients. Oral administration of cilostazol may be a novel therapeutic option for patients with bronchial asthma, for whom cough is an especially troublesome symptom. This is the first report demonstrating the efficacy of PDE 3 inhibition in view of cough reflex sensitivity in elderly asthmatics. Further studies are required to investigate the role of other PDE isozymes in airway cough reflex sensitivity in bronchial asthma.

## Abbreviations

cyclic-AMP = adenosine 3' 5'-cyclic monophosphate; CVA = cough variant asthma; FEV1 = forced expiratory volume in one second; FVC = forced vital capacity; GSEM = geometric standard error of the mean; PDE = phosphodiesterase; PGD2 = prostaglandin D2; PGE2 = prostaglandin E2; PGF2α = prostaglandin F2α; TNF-α = tumor necrosis factor-α; TxA2 = thromboxane A2; TxB2 = thromboxane B2.
